# Key Considerations When Developing and Implementing Digital Technology for Early Detection of Dementia-Causing Diseases Among Health Care Professionals: Qualitative Study

**DOI:** 10.2196/46711

**Published:** 2023-08-22

**Authors:** Sarah Wilson, Clare Tolley, Riona Mc Ardle, Emily Beswick, Sarah P Slight

**Affiliations:** 1 School of Pharmacy Newcastle University Newcastle upon Tyne United Kingdom; 2 Translational and Clinical Research Institute Newcastle University Newcastle upon Tyne United Kingdom

**Keywords:** qualitative, health care professional, early detection of disease, dementia, digital technology, early detection of dementia, digital health, health care worker, digital tool

## Abstract

**Background:**

The World Health Organization (WHO) promotes using digital technologies to accelerate global attainment of health and well-being. This has led to a growth in research exploring the use of digital technology to aid early detection and preventative interventions for dementia-causing diseases such as Alzheimer disease. The opinions and perspectives of health care professionals must be incorporated into the development and implementation of technology to promote its successful adoption in clinical practice.

**Objective:**

This study aimed to explore health care professionals’ perspectives on the key considerations of developing and implementing digital technologies for the early detection of dementia-causing diseases in the National Health Service (NHS).

**Methods:**

Health care professionals with patient-facing roles in primary or secondary care settings in the NHS were recruited through various web-based NHS clinical networks. Participants were interviewed to explore their experiences of the current dementia diagnostic practices, views on early detection and use of digital technology to aid these practices, and the challenges of implementing such interventions in health care. An inductive thematic analysis approach was applied to identify central concepts and themes in the interviews, allowing the data to determine our themes. A list of central concepts and themes was applied systematically to the whole data set using NVivo (version 1.6.1; QSR International). Using the constant comparison technique, the researchers moved backward and forward between these data and evolving explanations until a fit was made.

**Results:**

Eighteen semistructured interviews were conducted, with 11 primary and 7 secondary care health care professionals. We identified 3 main categories of considerations relevant to health care service users, health care professionals, and the digital health technology itself. Health care professionals recognized the potential of using digital technology to collect real-time data and the possible benefits of detecting dementia-causing diseases earlier if an effective intervention were available. However, some were concerned about postdetection management, questioning the point of an early detection of dementia-causing diseases if an effective intervention cannot be provided and feared this would only lead to increased anxiety in patients. Health care professionals also expressed mixed opinions on who should be screened for early detection. Some suggested it should be available to everyone to mitigate the chance of excluding those who are not in touch with their health care or are digitally excluded. Others were concerned about the resources that would be required to make the technology available to everyone.

**Conclusions:**

This study highlights the need to design digital health technology in a way that is accessible to all and does not add burden to health care professionals. Further work is needed to ensure inclusive strategies are used in digital research to promote health equity.

## Introduction

Dementia is a progressive neurodegenerative syndrome that causes deterioration in cognitive function beyond normal aging [1]. In the United Kingdom, approximately 900,000 people were reported to be living with dementia in 2021 [2]. The cost of dementia in the United Kingdom reached £34.7 billion (US $44.32 billion) in 2019 and is estimated to reach £94.1 billion (US $120.18 billion) by 2040 due to mounting health and social care costs [3]. With numbers projected to increase, the United Kingdom’s health and social care secretary announced a major conditions strategy in 2023, which aimed to alleviate pressure on the UK health system by harnessing digital technology (defined as electronic tools, systems, devices, and resources that generate, store, or process data) and innovation to enable earlier diagnosis [4]. The current dementia diagnostic process used by the National Health Service (NHS) in England requires a multidisciplinary approach for accurate diagnosis [5]. The recommended process involves conducting cognitive assessments to evaluate symptoms, using neuroimaging techniques to detect potential structural changes associated with dementia, and relying on clinicians’ judgment to make a final diagnosis [5]; these tests are dependent on available resources. The diagnostic process usually begins once clinical symptoms become apparent, which can be years after neuropathological changes such as amyloid plaques have developed [6]. There is a clear need for an inexpensive, scalable, easy-to-use technique to detect dementia-causing diseases (diseases that affect memory, thinking, and the ability to perform daily activities) at an earlier stage so they can prepare for the future and consider changes to their lifestyle in a way that could possibly reduce their risk.

Recently, there has been interest in using inexpensive, minimally invasive, scalable digital technologies such as wearables and smartphones to help detect and monitor digital biomarkers associated with the early stages of the disease [7]. Once the technology has detected these subtle changes, artificial intelligence (AI) can predict the likelihood of the user developing mild cognitive impairment and dementia, with improved sensitivity and specificity compared to traditional methods such as cognitive tests [8]. Different modalities could be measured and monitored remotely, for example, subtle changes in gait [9], speech [10], and cognition [11], which are all associated with the early stages of dementia-causing diseases. This approach might provide a more ecologically valid assessment due to the increased use of technology in society and the ability to capture data during daily activity [11]. Multiple research projects, including the Early Detection of Neurodegenerative (EDoN) diseases initiative [7] and Remote Assessment of Disease And Relapse—Alzheimer’s Disease (RADAR-AD) project [12], are now exploring the suitability of technology to detect and remotely monitor clinical and physiological factors (ie, digital biomarkers) associated with prodromal dementia-causing diseases.

Despite the potential benefits of using digital technology for the early detection of dementia-causing diseases, the development of clinically feasible technology is still in its infancy. To aid the development process, it is important to understand what challenges one might face when implementing these digital technologies in the NHS. Ethical concerns such as the capacity for postdetection management and the availability of effective interventions will need to be considered due to a lack of effective disease-modifying treatments for early-stage dementia [13,14]. Previous research has highlighted the benefits of involving end users, including patients, members of the public, and health care professionals, from the earliest stages of intervention development [15]. However, NHS health care professionals’ opinions and perspectives on the early detection of dementia-causing diseases using digital technology have not yet been explored. Identifying the potential challenges to implementation and concerns raised by health care professionals will help to inform the future development and implementation of digital technology and also support the National Institute for Health and Care Excellence (NICE) approval process [16]. There has yet to be a digital technology approved in the United Kingdom for early detection.

As part of the global EDoN initiative, we explored the perspectives of patients and the public [17] on using different tools for the early detection of neurodegenerative disorders. We sought to build on this prior work by exploring another group of end users, specifically health care professionals. This study aimed to gather health care professionals’ perspectives on the key considerations of developing and implementing digital technologies for the early detection of dementia-causing diseases in the English NHS.

## Methods

### Participants

Participants were recruited through various NHS clinical networks, such as the Future NHS collaboration platform (connecting individuals working in NHS health and social care), Faculty of Clinical Informatics (including health and social care qualified individuals working in the area of informatics across the United Kingdom), and British Geriatrics Society (multidisciplinary health care staff catering for the health care needs of older people). A snowball sampling approach was also used, which involved recruiting participants through existing participant networks [18]. Study information was distributed via email, social media posts, and newsletters. Interested participants then contacted the lead researcher to provide consent and schedule an interview. Health care professionals (any individual who provides health care treatment and advice based on formal training and experience) with patient-facing roles working in primary or secondary care settings in the NHS were eligible to take part. This included health care professionals with different levels of qualification or previous experience. Health care professionals did not have to be dementia specialists but were involved in a patient-facing role that could involve the initial referral, diagnosis, or after care of individuals with neurodegenerative diseases.

### Data Collection

The interview topic guide was designed by the research team and piloted by 2 general practitioners (GPs) with experience in managing individuals at different stages of receiving a dementia diagnosis (see Multimedia Appendix 1). The topic guide was further refined to improve the clarity of questions, and additional prompts were added to explore the areas of interest to the research team in further depth. These areas included participants’ experiences of the current dementia diagnostic practices, views on the early detection of dementia-causing diseases (particularly via screening), use of digital technology to aid early detection, and the potential challenges of implementing digital tools into the health care system (available on request). All interviews were conducted via video call by a trained researcher (SW), lasted 30 to 45 minutes, and were audiorecorded using the videoconferencing software: Zoom (Zoom Technologies, Inc or Microsoft Teams (Microsoft Corp) depending on the participants’ preference. Recordings were transcribed verbatim by a transcription company (University Transcriptions) and checked for accuracy.

### Data Analysis

Transcripts were uploaded to NVivo (version 1.6.1; QSR International) and analyzed using an inductive framework approach, a form of codebook thematic analysis [19,20]. Inductive thematic analysis is the process of identifying reoccurring patterns in the data to create concepts and themes without using any preconceptions or pre-existing framework [19]. The 5 main stages of the framework approach were followed, starting with familiarizing with the data by rereading the transcripts before generating initial codes. Codes were then grouped into core concepts (a central organizing concept, which holds associated shared meaning patterns, ie, themes), forming the analytical framework, which was then applied across the whole data set. Data were charted into the framework matrix (rows [cases], columns [codes], and “cells” of summarized data) to help summarize the large quantity of data collected. This further enabled our multidisciplinary team to review the analysis and interpretation of the data to provide a richer understanding of clinicians’ experiences and perspectives [20,21]. The flexibility of the framework approach allowed us to explore specific project aims (eg, gather health care professionals’ perspectives on the key considerations of developing and implementing digital technologies for the early detection of dementia-causing diseases in the NHS while being open enough to uncover new meaning and unexpected concepts) [20,21]. The lead researcher (SW), with contributions from other authors (CT and SPS), moved backward and forward between the data, comparing data, and evolving explanations. Further discussion and refinement of the central concepts and themes with coauthors (CT, SPS, and RMA) was conducted to ensure the themes were reflected by the data. The authors were satisfied when data saturation was reached before participant recruitment eased.

### Trustworthiness

Trustworthiness was achieved by ensuring the credibility, transferability, dependability, and confirmability of the study [22]. We followed guidance outlined by Nowell et al [23] and used strategies such as piloting the interview topic guide with 2 GPs, triangulation of participants (ie, a range of different health care professionals), and having regular research team meetings at the analysis stage. To address credibility, we included peer debriefing to provide an external check on the research process and interpretation of the data. Transferability, which really only concerns a case-to-case transfer, was achieved by providing a rich description of the study methods so that those who seek to transfer the findings to their own site can judge transferability. To achieve dependability, we ensured that the research process was logical, traceable, and clearly documented. According to Lincoln and Guba [22], confirmability is established when credibility, transferability, and dependability are all achieved. We also included the reasons for theoretical, methodological, and analytical choices throughout, so others can understand how and why decisions were made.

### Ethics Approval

Ethical approval was granted by the Newcastle University Faculty of Medical Science Ethics Committee (17031/2021). Participants were not remunerated after taking part in the study. Voluntary informed consent was obtained in writing from the participant before the interview began. Participants were also asked to give verbal consent at the start of the interview for the discussion to be audiorecorded. The recordings were transcribed verbatim, deidentified (eg, names were removed), and anonymized by placing a unique participant identification number on each electronic file. Direct quotations used in the analysis are not identifiable by name but do correspond with the participant’s unique identification number.

## Results

### Overview

**Figure 1:**
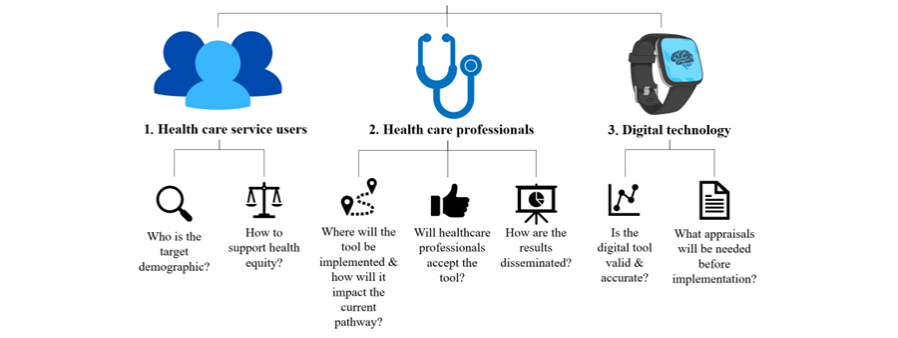
Summary of core concepts describing the key categories that need consideration when developing and implementing digital technology for the early detection of dementia-causing diseases.

Eighteen individuals agreed to participate (see Table 1), including 11 GPs, 2 mental health nurses, 1 professional with a joint role of mental health nurse and family care coordinator, 1 mental health care coordinator, 1 dementia specialist nurse, 1 psychiatrist, and 1 community psychiatric nurse. Ten participants were female and 8 were male and had a range of between 7 and 45 years of experience working in the NHS. Participants were based in various regions in England, including the Northeast, Northwest, Southeast, Southwest, West Midlands, and East Midlands. We identified several key considerations that are relevant to health care service users, health care professionals, and the digital health technology itself (see Figure 1 and Table 2). Each core concept and its associated themes are described in detail below using direct quotes from the interviews. To ensure participant confidentiality, participant names have been replaced with their participant ID (eg, P1: participant #1).

**Table 1:** Participant characteristics.

Participant ID	Role	Setting	Sex	Years in the NHS^a^	Professional interest
P1	GP^b^	Primary care	Male	12	Mental health and dementia
P2	GP	Primary care	Female	18	Frailty and dementia
P3	GP	Primary care	Male	31	Management, commissioning, and digital health
P4	GP	Primary care	Male	26	Mental health
P5	GP	Primary care	Female	27	Frailty and dementia
P6	GP	Primary care	Female	19	Older people’s health and frailty
P7	GP	Primary care	Male	10	Dementia with personal experience of caring for someone with dementia
P8	GP	Primary care	Male	16	Dementia
P9	GP	Primary care	Female	11	Dementia
P10	Mental health nurse	Secondary care	Female	15	Mild cognitive impairment
P11	Mental health nurse	Secondary care	Female	22	Dementia
P12	Dementia specialist nurse	Secondary care	Male	16	Mental health
P13	GP	Primary care	Female	31	Mental health with personal experience of caring for someone with dementia
P14	GP	Primary care	Male	35	Dementia and learning disabilities
P15	Psychiatrist	S	Female	16	Mental health
P16	Mental health nurse and family care coordinator	Secondary care	Female	11	Mental health
P17	Community psychiatric nurses in older person’s service	Secondary care	Male	45	Physical and mental health in older people
P18	Care coordinator in a community mental health team	Secondary care	Female	7	Dementia

^a^NHS: National Health Service.

^b^GP: general practitioner.

**Table 2:** Summary of core concepts and themes

Core concept and theme		Description
**Health care service users**		
	Target demographic	Identifying the group most suitable for early detection of dementia-causing diseases.
	Health disparities	Consideration for digital exclusion and those with additional needs.
**Health care professionals**		
	Implementation and impact on clinical practice	The setting to implement the digital technology in current clinical practice, the potential impact the tool could have on the health care system, and additional resources needed to support successful implementation.
	Acceptance	Factors that might affect health care professionals’ acceptance to use the technology.
	Dissemination of results	Interpreting, understanding, and explaining the results to health care users.
**Digital health technology**		
	Accuracy and validity	Evidence of validity, comparison to other tools, and the need to incorporate an individual’s medical and family history into the technology.
	Technology appraisals	Necessary appraisals and approvals needed before implementation.

### Considerations for Health Care Service Users

Health care professionals expressed mixed opinions on who should be screened for an early detection of dementia-causing diseases, with some suggesting keeping the eligibility criteria “as broad as possible” (P6, GP) to avoid missing a “proportion of the population who are actually possibly [at] a much higher risk [but] aren’t as engaged or as involved with their healthcare” (P6, GP) as other individuals. However, others highlighted “how the amount of resource to go into screening people under the age of 65 [and are asymptomatic]...would seem disproportionate to the number of cases picked up” (P2, GP). Some interviewees instead recommended targeting “patients who have higher risk factors” (P3, GP), for example, those with a family history of dementia or long-term medical conditions.

Health care professionals also raised how the digital tool would need to be available to everyone to ensure that we are “not excluding people who already are in digital poverty” (P5, GP). This stemmed from their experiences of caring for populations where “most of our people [health care service users] don’t have a smartphone” (P18, care coordinator), which would in turn limit their use of certain apps. The mental health nurse and family care coordinator was also of the opinion that many of those who did own a phone “don’t know how to use their mobile phone” (P16, mental health nurse and family care coordinator), which may in turn contribute to digital exclusion. Concerns were also raised about those individuals with disabilities, such as learning disabilities or visual and hearing impairments, because they “may misinterpret what’s in front of them” (P17, community psychiatric nurses) or misunderstand what they are being asked to do. One health care professional reflected on how they nearly misdiagnosed a health care service user with dementia when the individual was actually depressed. In this particular case, the health care professional “couldn’t understand [the health care service user], we thought a lot of it was just confabulation but it was actually, she was trying to express how she was but in her native language” (P17, community psychiatric nurse); it was only when an interpreter was used that the health care professional understood the individual whose first language was not English and could make a more accurate diagnosis.

### Considerations for Health Care Professionals

Some GPs suggested that digital technology could be implemented in primary care and included as part of a screening campaign or an NHS annual health check. However, there were concerns about overburdening the NHS if the digital technology identified a large number of individuals who may be at higher risk and subsequently referred to secondary care:

Waiting times to go to memory clinic for people who have symptoms is already really quite significant. And if we’re now looking at screening people who don’t have symptoms, who then need a full formal assessment. And consideration of medications the waiting list for memory clinics would dramatically increase...unfortunately, we don’t have the workforce in secondary care to manage that demand.

P6, GP

A mental health nurse and family care coordinator also highlighted how secondary care “only really get referrals once someone is showing signs [of cognitive decline]” (P12, dementia specialist nurse). Therefore,

If someone was high risk [of a dementia causing disease] the GP wouldn’t let us know because we can’t do anything with that information....We couldn’t deal with reviewing information for lots of people because it’s not relevant to our service but more for the GP they could maybe just get them in every six months to review.

P16, Mental health nurse and family care coordinator

Many health care professionals working in secondary care shared this view, with another suggesting that brain health centers might be a more suitable place to implement such digital tools, as they combine “clinical and research methods and techniques to sort of assess people with suspected dementia” (P15, psychiatrist).

Health care professionals also wanted to know “who takes responsibility” (P2, GP) for assisting health care service users with digital technology (ie, showing them how to use the technology). Some felt that the task might be very time intensive and that there may be a need for additional health care professionals, such as support workers or health care assistants, to assist with this set-up process.

Many health care professionals were also concerned about having suitable space to deliver a new service, since “rooms are at a premium in general practice” (P13, GP). One participant highlighted how extra resources would be needed since GPs are not currently “paid from the CCG [clinical commissioning groups] to offer that service [early detection]” (P11, mental health nurse). Additional resources would also be required to fund future treatments for individuals, bearing in mind that “the new drugs are extremely expensive” (P6, GP). Furthermore, additional psychological and practical support services might need to be offered to those individuals identified at higher risk, as it could “affect their [life] insurance” (P2, GP) and employability. One GP was concerned that the early detection tool could create a “tidal wave of anxious people that you [health care service] can’t manage” (P3, GP). Other participants questioned whether health care professionals have the capacity to take this on “since people [health care professionals] are very busy, or feel stressed already, you’re [some health care professionals] just not in a place where you can maybe take on new things” (P18, care coordinator).

Health care professionals suggested marketing the tool as exciting so that “it piques people’s interest” (P13, GP) and involving clinicians in the implementation process to ensure they feel “in some way part of that, not just that somebody’s told you it’s a good idea” (P18, care coordinator). They also highlighted how the digital technology must be usable: “you can make the most perfect thing in the world, but if no one’s going to use it and it’s not user-friendly, then people won’t do it” (P9, GP). Training with ongoing support was also viewed as crucial to avoid the tool “just sitting in the cupboard” (P12, dementia specialist) becoming an “expensive white elephant” (P5, GP). Some health care professionals also wanted to see evidence of effective intervention options for individuals who were identified as at higher risk because “if you can’t do anything about it then what’s the point” (P1, GP).

Health care professionals felt the output from the digital tool should be easy to interpret and “integrate with our computers [in the NHS system]” (P5, GP). Many health care staff also felt strongly about being able to understand how the AI algorithm derived its output or risk prediction score and the predictors or indicators that were used, for example, if one of the indicators was like people having a poor sleep pattern and then we could follow that up...what is it about the sleep pattern that makes you [patient] at risk?” (P18, care coordinator). This understanding “would [also] help you explain it better to the patient. (P15, psychiatrist), as the patient “may be quite sceptical if you then couldn’t say, well [it is] based on x, y and z variables, and that’s how we came up with this score” (P15, psychiatrist).

Health care professionals suggested having good visual aids to help patients understand the results. This was felt to be particularly important if a future tool generated a risk score, stating, “in the next 10 years the chance of you developing a dementia is X percent” (P4, GP), because “people’s understanding of risk varies so much individually” (P6, GP). Health care professionals described how health care service users will also need to be informed about “what they need to do to keep their risk low” (P5, GP), such as “adopting a healthy lifestyle” (P4, GP). However, “in quite a deprived area, lifestyle changes aren’t always taken up as we would like them to” (P6, GP), so dissemination and design of any intervention will need to be considerate of existing health disparities to support health equity.

### Considerations for Digital Health Technology

Health care professionals described the importance of having evidence to show “the sensitivity and specificity” (P4, GP) of the tool, “how accurate the digital test is. Is it likely to pick up a positive, if it is positive? Or, can you get false negatives?” (P4, GP), and how the results could be affected if the digital technology was “not worn properly” (P12, dementia specialist). Some health care professionals felt that the digital technology should be “validated in the population [it is intended to be used in] and validated against what we do now, to show that it’s better or superior or comparable” (P14, GP) to what they currently do. Some questioned whether it was possible to replicate current paper-based tools such as the ACE-III (Addenbrooke’s Cognitive Examination) where,

You’ve got to get people to write things, draw things, you’ve got to get them to do tasks, so you can’t actually do that on a computer, you know, things like drawing and that on a computer, it’s not the same.

P17, Community psychiatric nurse

Health care professionals did recognize the advantages of collecting real-time data “in their [health care service users’] own home and, [if] the results are validated and it can be fed to a health care professional that would be excellent because that is probably likely to be real time data which might be more relevant than an artificial questioning in a surgery [British English term for clinic]” (P1, GP). Several GPs described how it would be useful if the tool could monitor neurodegenerative syndrome progression over time; for example, “has this been just a slow, steady decline or have there been big drops, which helps build what kind of dementia I think we’re looking at, or if it’s acute or not really a dementia” (P9, GP).

Health care professionals felt it could be important for family members or caregivers to “be able to input into that [process and] to give their opinion” (P16, mental health nurse and family care coordinator) as they can provide a “wealth of information” (P16, mental health nurse and family care coordinator) around their relative’s decline.

Health care professionals felt that it was important to obtain all the necessary approvals prior to implementation but recognized how very few screening tools have been approved by NICE. There was also the issue that even if a digital early detection tool was included in NICE guidelines, adherence is “very much clinician dependent” (P10, mental health nurse) and appeared to also rely on obtaining the necessary resources,

Services that I’ve worked in the past, we followed the NICE guidance more closely and offered imaging to most people.... The reality in our [current] service is that we are a bit more cautious about who we request imaging for.

P15, Psychiatrist

## Discussion

### Principal Findings

This study is the first to report health care professionals’ key considerations in the development and implementation of digital technologies for the early detection of dementia-causing diseases in the NHS. Health care professionals described how the digital tool should not exacerbate digital exclusion, discussed the need for scientific evidence to show that the technology is accurate and reliable, and highlighted the importance of appropriate training and support for health care service users and health care professionals. Finally, participants also shared concerns about the risk of such tools overburdening the NHS in terms of creating additional workload for health care professionals and requiring further resources to support implementation and use, such as additional staff.

It is important to promote digital inclusivity when designing and implementing digital technology in health care. The WHO [24] and NICE [16] have highlighted how measures should be taken to address inequities in access to digital technology so that further inequity is not perpetuated in terms of accessing health information and services. They also stressed the importance of having health interventions that are “inclusive by design” [16]. However, some of the demographic factors associated with developing dementia, such as age, ethnicity [25], and educational attainment [26], put individuals at greater risk of digital exclusion. This may be due to a combination of differences in access, use, motivation [27], and reliability of sensors used in digital technology across different demographics [28], resulting in widening health inequalities, and in some cases, failure of interventions to result in demonstrable health benefits in certain populations [29,30]. Strategies to reduce digital health inequities must be adopted, for example, by providing the option of different language settings and basic digital literacy skills training.

Participants in this study highlighted the importance of identifying an appropriate clinical setting to introduce an early detection tool. The setting would have to be sufficiently resourced in terms of staff and technology to perform the tests and should also offer support to those who are perceived to be at higher risk [13]. New initiatives such as Brain Health Clinics (an integrated research and clinical environment dedicated to improving the assessment and diagnosis of memory problems including earlier diagnosis) were suggested as a possible setting where such tools could be implemented. These sites have been established in Scotland and certain places in the United Kingdom by the government (in Scotland) and research grants (in England) and contribute toward researching personalized risk profiling, targeted risk reduction and prevention, early disease detection, equity of access, and harnessing comprehensive data to assist in clinical decision-making [31,32]. Brain health clinics located in Scotland hold a rational and scientific basis for a national care pathway [31], but brain health clinics in England do not have this in place. The level of development of these sites may affect health care professionals’ perspectives of where digital technology for early detection of dementia-causing diseases might be best placed.

Training to support the correct use of digital technology and interpretation of the results was recognized as crucial to the success of implementing new digital tools into practice. Tucci et al [33] also found explainability, transparency, integrability, and usability are key factors affecting health care professionals’ trust and acceptance of AI in health care practices [33], echoing the concerns raised by health care professionals in this study. Health care professionals need to be able to trust the outputs of any computerized decision support tool, and information should be easy to interpret and delivered efficiently, for example, by integrating with existing computerized software to support clinician workflow [33-35]. The output should also be tailored to the patient [16], which will require better availability and use of patient data within and across systems. While acknowledging the challenges of processing sensitive patient information, the WHO promotes syntactic and semantic interoperability with WHO standards as a cornerstone of health information to enable sharing of information [36].

A further important area raised in our study was the ethical concerns around delivering or receiving an early detection of dementia-causing diseases. This echoed the results of a recent study that explored the views of Dutch physicians on the early diagnosis of Alzheimer disease and found that diagnosis “must have a function, or it must be able to contribute to a better happiness, so to speak” [37]. Health care professionals’ fears may also relate, more generally, to the premature or incorrect labeling of an individual with dementia, with the potential psychological effects that this could cause [38]. The tool will have to go through rigorous testing to ensure that it is sensitive and specific, so as to alleviate professionals’ concerns of false results but also to reassure members of the public if used as part of a national screening program [39,40]. Recent developments of drug interventions, such as the monoclonal antibody lecanemab, have shown the potential to reduce physiological biomarkers in early Alzheimer disease, which in turn may increase the acceptance of early detection in the future [41]. Furthermore, there is a growing body of evidence showing behavioral and lifestyle changes can reduce the risk of developing dementia-causing diseases [42], which could be implemented as part of a holistic, early detection approach alongside any effective pharmaceutical intervention.

### Strengths and Limitations

A range of different professionals who had a substantial amount of experience working in the NHS were interviewed in this study. These participants performed a range of different patient-facing roles in different health care settings. Data were collected until thematic saturation was achieved, and a robust data analysis conducted to ensure the trustworthiness of our findings [22,23]. However, many of these participants had a professional interest in mental health, older people’s health, dementia, mild cognitive impairment, or digital health, which may have influenced their perspectives.

Further research should explore the opinions of stakeholders, such as commissioners and policymakers, to help identify other specific challenges that may impact the implementation process. It is also clear that the development of novel digital technologies must promote inclusivity and ensure intervention options are accessible to all. Feasibility testing of these newly developed technologies will be required to further enhance and ensure that these products are both relevant and useful [43]. Research is also needed to understand how health care professionals and health care users would like computer-generated information to be presented to them; this would help enhance trust and understandability of any outputs.

### Conclusions

This study highlights the key considerations when developing and implementing digital technologies for the early detection of dementia-causing diseases. This information will help guide future work around the current needs of health care professionals from the technology, and the challenges of implementing in the NHS. It is important that those involved in the design and development of novel digital technologies continue to work closely with users, health care professionals, and stakeholders, including policymakers, regulatory bodies, and commissioners to aid successful implementation.

## Appendix

Multimedia Appendix 1 Interview guide.

## Abbreviations

ACE-III: Addenbrooke’s Cognitive Examination
AI: artificial intelligence
EDoN: Early Detection of Neurodegenerative
GP: general practitioner
NHS: National Health Service
NICE: National Institute for Health and Care Excellence
RADAR-AD: Remote Assessment of Disease And Relapse—Alzheimer’s Disease
WHO: World Health Organization

## Data Availability

The data sets generated and analyzed during this study are not publicly available due to a potential breach of participant confidentiality but anonymized data sets may be available from the corresponding author on reasonable request.
