# Supplementary material for: Key Considerations When Developing and Implementing Digital Technology for Early Detection of Dementia-Causing Diseases Among Health Care Professionals: Qualitative Study
Source: J Med Internet Res. 2023 Aug 22;25:e46711. doi: 10.2196/46711 (PMC10481214; doi:10.2196/46711)
Supplement: Multimedia Appendix 1 [file jmir_v25i1e46711_app1.docx]

**Clinician interview topic guide**

I would like to start by thanking you for making the time to participate in today’s interview. I’m Sarah Wilson, a Research Assistant at Newcastle University and I would like to understand your approach to assessing and managing patients with neurodegenerative diseases, more specifically those on the dementia care pathway. Is there anything you would like to ask me before we start?

Are you still happy for this interview to be recorded? (Confirm receipt of signed consent form)

1. To start with, would you mind if I asked you a few questions about yourself please?

- *How many years have you been working in the NHS? More specifically in general practice?*
- *What is your position within your practice?*
- *Where is your practice based? And what is the typical demographics of the patients that you see?*
- *Have you got any special interests within general practice?*

1. We’ll move on now to patients with neurodegenerative diseases… ..... What is your understanding of neurodegenerative diseases? *Have you got much experience in this area?*
2. For the rest of the questions, we will focus specifically on Dementia and MCI…
3. How would you assess a patient’s cognitive function and the stage they are at e.g., early or late stages of dementia/ MCI?
   - ***When*** *would you initiate this?*
   - ***What*** *methods would you use?* Pros/cons of using these methods?
   - *Do patients need any support to use the methods/tools for assessment? –* ***Who*** *would provide this?*
   - *Is there a* ***cost*** *attached to using these tools?* ***Who*** *covers this cost?*
4. Have you or would you consider using digital technology such as wearable sensors or apps to assess a participant’s cognitive function?

*If so, which apps have you used?*

1. If a patient appears to show signs of a dementia or MCI what are the next steps on the care pathway?
   - ***What*** *information guides your judgment on what to do next?*
   - *Could digital technology play a role in the monitoring cognitive function of these patients?*
   - *What additional information might be useful for you to know (e.g., technology may provide)?*
2. Would you suggest any lifestyle changes or treatments to patients that show signs of a neurodegenerative disease?
   - *What information guides your judgement?*
   - *Would you suggest anything different for those shown signs of dementia?*

If we think about the pre-clinical stages of MCI or dementia (so before any symptoms become apparent),

1. What are your thoughts on screening patients for pre-clinical stages?
   - *Using digital technology like wearable sensors or smartphone apps to be able to detect pre-clinical stages of MCI or dementia?*
   - *What impact might detecting pre-clinical stages have on patients and on general practice?*
2. What information would be useful to get from a screening tool?

- *Categorise patients at high, medium or low risk of developing dementia or other neurodegenerative diseases?*
- *How would you manage these different categories of patients?*

1. In terms of implementation - how would you go about implementing a screening tool in your GP practice*?*

- *Which patients would you screen? Target specific groups? Or all patients e.g., include in the general health check (over 40s)?*
- *Who would be the best person in your practice (GP, nurse, etc) to carry out the screening?*
- *What challenges might you face? Volume of patients? Staff training?*

That was the final question. Thank you for taking the time to participate. We are keen to seek the perspectives of a range of practice staff in different practices. Any help that you can provide in facilitating this would be helpful. Do you know of any healthcare staff networks that we could approach or any contacts that might be happy to participate in an interview?

Thank you
